# Typology of murder-suicides in Berlin according to a longitudinal study based on autopsy files

**DOI:** 10.1007/s12024-021-00360-6

**Published:** 2021-02-09

**Authors:** Milan Zimmermann, Michael Tsokos

**Affiliations:** 1 Center of Neurology, Department of Neurodegeneration and Hertie-Institute for Clinical Brain Research, University of Tuebingen, 72074 Tübingen, Germany; 2 German Center for Neurodegenerative Diseases (DZNE), University of Tuebingen, 72074 Tübingen, Germany; 3 Department of Forensic Medicine, Charité-Universitätsmedizin Berlin, 10117 Berlin, Germany; 4 City Institute for Forensic Medicine, Berlin, Germany

**Keywords:** Murder-suicides, Homicide-suicides, Suicides, Criminal law, Depression, Psychopathology

## Abstract

Murder-suicides are defined as the murder of at least one person and the suicide of the offender following the murder. The intention to commit suicide must be primary. In most cases, a male offender kills a female victim after a separation. The current analysis was the first analysis of the typology of murder-suicides in Berlin. We analyzed the autopsy files of the Institute for Forensic Medicine of the Charité University Medicine Berlin and of the City Institute for Forensic Medicine Berlin. We performed descriptive and statistical analyses of cases between 2005 and 2013. We identified 17 murder-suicides. All 17 offenders were male, and 20 of the victims (90%) were female. The offenders used firearms in the majority of the cases. In seven cases, the victims and offenders were at least 80 years old. The average age of the offenders was 63 years. Disease was the motive in 6 cases involving older offenders. Our study might support the development of prevention strategies. In this regard, it is important to build a database for murder-suicides in Germany and other countries, to formulate a uniform definition of murder-suicide, to carry out nationwide interdisciplinary studies on this topic and to improve the existing health care structures, especially for older adults and people with depression.

## Introduction

A murder-suicide is defined as the murder of at least one person and the suicide of the offender following the murder. The intention to commit suicide must be primary. The time span between the murder and the suicide as part of the definition of a murder-suicide is controversial. Marzuk et al. [1] defined the maximum time span as one week. The inclusion of a time span in the definition of a murder-suicide might help differentiate between murder-suicide and suicide out of remorse [2]. Most analyses of murder-suicides refer to newspaper articles or forensic files because in most countries, there is no central register for murder-suicides [3]. Data from the USA show that the incidence of murder-suicide lies between 0.2–0.3/100,000/year, which means that 1000–1500 deaths per year occur as part of a murder-suicide [1, 3]. A study based on the data from the National Violent Death Reporting System found 144 cases of murder-suicides, with 164 murders and 144 suicides in 2004 [4]. Liem et al. [5] found that 176 people per year, accounting for approximately 4% of all homicides, die due to a “murder-suicide”. Marzuk et al. [1] found that the offender was male in 94% of the cases and that the victims were female in 85% of the cases. In the study by Bossarte et al. [4], 82.7% of the offenders used firearms and 6% used sharp weapons when committing the homicides. Approximately 65% of the victims were married to the offender or lived in a civil partnership with them [1]. Most offenders were between 40 and 50 years old and most were a few years older than their victims [3]. In most cases, the murder and the suicide occur in the same location [4].

Female offenders of a murder-suicide usually kill their children (48.6%), whereas male offenders usually kill their intimate partner [6]. Domestic violence occurred in 25% of cases before the murder-suicide [3]. Employment was not found to be protective against murder-suicide, as 77% of the offenders had at least one job [3]. Approximately 39% of the offenders had depression [7].

Suicide notes were found in approximately 70% of all cases [8].

The most popular system of categorization, which was introduced by Marzuk et al. [1], is based on possible motives for the murder-suicide. They classified murder-suicides according to the following categories: “amorous jealousy”; “mercy killing”; “altruistic or extended suicides”; “family, financial or social stressors”; and “retaliation”. The category “amorous jealousy” includes murder-suicides in which an offender kills his or her partner after a separation or the announcement of an impending separation. In such cases, perhaps the offender considers the victim to be an extension of himself. “Mercy killings” describe cases of older offenders killing their partners because of declining health or advanced disease. “Pseudo-altruistic” motives might play a role in cases of female offenders killing their children. They might not want them to be completely at the mercy of, for example, a former partner, or to face an unfavorable future [1].

Malphurs and Cohen stated that 70.5% of murder-suicides can be assigned to the category “spousal/consortial”, 10.5% to the category “infanticides”, 8.7% to the category “extrafamilial murder suicides” and 6.5% to the category “familicides” [3].

Another categorization of motives was discussed by Joiner [2] and Adhia et al. [9]. The theory of a “perversion of virtue” involves the misapplication of virtues such as mercy, justice, duty and glory. According to this theory, the offender would consider his offence to be moral [2].

In the current analysis, we analyzed the typology of murder-suicides in Berlin for the first time. This might help support the development of prevention strategies to decrease the frequency of murder-suicides in the future.

## Methods

### Materials and methods

We analyzed the autopsy files of the Institute for Forensic Medicine of the Charité University Medicine Berlin and of the City Institute for Forensic Medicine Berlin. We considered cases occurring in an 8-year time interval between 2005 and 2013. The autopsy files comprised both autopsy reports and toxicological analyses. Some files also included partial information about the first police investigations, trials, newspaper articles and suicide notes. A “case” was defined as all deaths linked with a causal relationship. A limitation on the time span between the homicide and the suicide was not applied. A case was considered a murder-suicide if the suicidal intention was primary. Double suicides, in which both participants commit suicide on their own in a time-bound and situational context, were not considered. Dual suicides in which both individuals had suicidal intentions but the fatal action was taken by only one person were considered murder-suicides. These included cases in which the participants supposedly mutually agreed to die together, but only one of the participants committed both the homicide and the suicide.

### Statistics

Statistical analysis was performed using IBM SPSS Statistics (IBM Corp. Released IBM SPSS Statistics for Macintosh, Version 22.0. Armonk, NY: IBM Corp.). For the analysis of the demographic data (age of offender and victim), the Mann–Whitney U-test and median test were used. For comparisons between age groups, a chi-squared test was performed.

## Results

### Frequency of murder-suicides

We found 17 cases fulfilling the criteria for murder-suicides. These cases involved the deaths of 17 offenders and 20 victims. Two victims were severely injured, and one dog was shot and killed. There were three cases in 2005, zero in 2006, two in 2007, three in 2008, one in 2009, one in 2010, three in 2011, three in 2012 and zero in 2013.

We found 15 cases involving one victim, one case involving two victims and one case involving three victims.

### Sex of victims and offenders

All offenders were male. The victims were female adults in 16 cases. Two underage female and two underage male individuals also died. The two severely injured individuals mentioned above were underage males.

### Age at the time of the murder-suicide

**Fig. 1:**
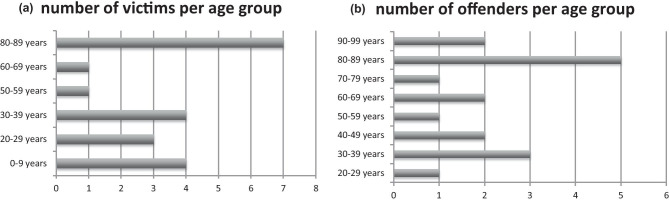
Number of victims (**a**) and offenders (**b**) per age group. 8 out of 20 victims were at least 60 years old. 10 out of 17 offenders were at least 60 years old

Figure 1 shows the distribution of victims (Fig. 1a) and offenders (Fig. 1b) among the different age groups. Most victims and offenders were between 80 and 89 years old (7 victims and 5 offenders). The mean age of the victims was 47.3 years, and the median was 36 years. The mean age of the offenders was 63.1 years, with a median of 69 years. The offender was older than the victim in 15 of 17 cases; in two cases, the female victim was one year older than the offender. The mean age difference was 5.6 years (*p* = 0.091), and the median was 3 years (*p* = 0.417).

### Relationship

In 12 out of 17 cases (70.59%), those involved in the murder-suicide were married; one couple was living apart from each other. In 7 of the married couples, both partners were at least 80 years old. In three cases, the victims and offenders were former couples. One victim and one offender lived together and were involved in an intimate relationship but were not married.

### Domestic violence

In one case, autopsy revealed signs of previous domestic violence. In another case, the female victim lived in a women’s shelter prior to the murder-suicide. In a third case, there were several police investigations due to reports of domestic violence prior to the murder-suicide. In three more cases, we found that the offender had a criminal record.

### Crime scene

In 14 of the 17 cases, both the homicide and the suicide occurred in the same flat; in four cases, they occurred in the same room. Most cases (4) occurred in Berlin’s Charlottenburg-Wilmersdorf district.

### Time interval

Most cases (10) occurred between February and May. In 10 cases, the homicide and the suicide took place on the same day. In two cases, they happened on two different days.

In one case, both the homicide and the suicide occurred at the same time because just one shot killed both individuals. In three cases, the rectal temperatures of the victims and offenders were similar. In another case, a witness reported that the shots were fired in rapid succession. In four cases, the suicide note revealed the possible time interval (25 min; 6.5 h; 12 h; 24 h).

### Causes of death

In many cases (40%), the victims died due to bullet wounds (8 out of 20 victims), including one child.

In three cases, death was caused by one shot to the head. In four cases, there were two bullet wounds; in one case, there were 5 bullet wounds. Five victims who died due to bullet wounds were over 50 years old. Other causes of death included a cut injury to the neck (31 years old), a stab injury to the neck (33 years old), intoxication combined with suffocation with a plastic bag (88 years old), suffocation with a plastic bag (2 years old), suffocation with a cotton diaper (82 years old), strangulation (88 years old and 22 years old) and suffocation due to CO_2_ intoxication using cartridges (see Table 1).

**Table 1:** Relationships, causes of death, weapons and motives in all 17 cases of murder-suicide. v: victim; o: offender

Case	Age and sex/ relationship	Cause of death (weapon)	Supposed motive
1	v: 31 (Female)	Exsanguination due to a neck cut (knife)	Jealousy due to an affair of his partner
	o: 36 (Male); Married		
2	v: 23 (Female)	Gunshot wound of the head (pistol); Victim with two wounds; Offender with one wound;	Separation
	o: 31(Male); Former relationship		
3	v :2 (Female)	One gunshot wound of the head (revolver); Offender held both heads together and shot once	The mother of the joint child wanted to separate from her partner
	o: 52 (Male): Father and daughter		
4	v: 36 (Female) o: 43 (Male); Partnership	v: Exsanguination due to a neck cut o: Exsanguination due to a neck cut and a cut to the breast (kitchen knife)	Unknown
5	v: 88 (Female) o: 93 (Male); Married	v: Intoxication in combination with suffocation (plastic bag); o: Hanging (electronic cable)	Disease of the female victim; imminent move to a nursing home
6	v: 82 (Female)	v: Suffocation (cotton diaper) o: Suffocation (two plastic bags)	Disease of the woman
	o: 84 (Male); Married		
7	v: 59 (Female)	v: Five gunshot wounds (heat, breast and abdomen) o: One gunshot wound (head) (firearm)	Separation
	o: 60 (Male); Former relationship		
8	v: 88 (Female)	v: Strangulation (scarf) h: Hanging (cord)	Disease of the victim
	o: 87 (Male)		
9	v: 85 (Female)	v: Gunshot wound (heard) o: Gunshot wound (head) (firearm)	Disease of the victim
	m: 88 (Male); Married		
10	v (1): 22 (Female) (w. 22 J.)	v (1): Strangulation (belt) v (2): Suffocation (plastic bag and cable ties) o: Suffocation (plastic bag)	Financial problems
	v (2): 2 (Female) o: 25 (Male); Former partnership and daughter of the victim		
11	v: 69 (Female)	v: Two gunshot wounds (head and neck) o: One gunshot wound (head)	Unknown
	o: 70 (Male); Married		
12	v: 86 (Female)	v: Strangulation o: Hanging (cord)	Disease of the offender
	o: 85 (Male); Married		
13	v: 33 (Female)	v: Strangulation (electric cable) o: Polytrauma (fall from a great hight)	Separation of the victim
	o: 36 (Male); Married		
14	v: 80 (Female)	v: Gunshot wound (head) o: Gunshot wound (head) (firearm)	Unknown
	o: 81 (Male); Married		
15	v: 86 (Female)	v: Two gunshot wounds (head and breast) o: One gunshot wound (head) (pistol)	Diseases of both victim and offender
	o: 90 (Male); Married		
16	v (1): 28 (Female) v (2): 3 (Male) v (3): 6 (Male) o: 69 (Male); Married; and joint children	Suffocation (CO2-cartridges)	Financial problems
17	v: 39 (Female)	v: Two gunshot wounds (thorax) o: Three gunshot wounds (thorax) (firearm)	Unknown
	o: 40 (Male); Married		

In most cases (47%), the offenders died due to bullet wounds (8 out of 17 suicides). In 7 cases, it was a shot in the head. In one case, there were three bullet wounds in the thorax area.

In six cases, the offenders were over 50 years old; in three cases, they were over 80 years old. Causes of death other than bullet wounds included a cut injury to the neck (36 years old), several cut injuries to the neck and the thorax (43 years old) leading to hemopneumothorax and the aspiration of blood, hanging (85 years old; 87 years old; 93 years old), suffocation under a plastic bag (25 years old; 84 years old), polytrauma due to falling from a great height (36 years old) and suffocation due to CO_2_ intoxication using cartridges (69 years old) (see Table 1).

In 13 out of 17 cases (76.5%), the victims and offenders died due to the same cause.

### Weapons

In 8 cases, a gun was used for both the homicide and the suicide. In two cases, a knife was used for both deaths. Other weapons used were an electric cable, a belt, plastic bags (cinched with a string or cable tie, among other means), a scarf, a cotton diaper and CO_2_ cartridges (see Table 1).

### Toxicological screening

The toxicological screening results in one offender revealed an alcohol concentration of 1.4 per mil (60 years old). The screening also showed evidence of the use of tramadol. One victim had an alcohol concentration of 0.5 per mil (85 years). There was no evidence of the use of other drugs prior to the murder-suicide. A two-year-old female victim was given diazepam prior to the murder. Her murderer (54 years old) also took diazepam prior to the murder-suicide. There was evidence of the use of diazepam in a 22-year-old victim and a 25-year-old offender who also took zolpidem prior to the murder-suicide. An 84-year-old offender most likely took the medication prescribed to his wife prior to death (clozapine and oxazepam).

### Suicide warning signs

In 6 cases, there were suicide warning signs prior to the murder-suicide.

### Suicide notes

In 8 cases (47.1%), there were suicide notes.

### Motives

In 13 out of 17 cases, we were able to determine the possible motive for the murder-suicide. In four cases, the motive was most likely the separation of a couple. In those cases, the offenders were 31, 36, 56 and 60 years old. In one case, the offender (36 years old) assumed that his wife had betrayed him. In 6 cases, disease played an important role (6 out of 13 cases; 46.2%). In four cases, the female victim had a disease (parkinsonism, dementia (2 cases), and prior stroke). In one case, there was evidence that the offender had a severe illness, and in one case, there was evidence that both the victim and offender had serious illnesses. All 12 victims and offenders were at least 80 years old and married.

Financial motives were likely in two cases of “family annihilation”, in which children were also killed. The offenders were 25 and 69 years old (see Table 1).

In two cases, there was evidence that a mutual agreement was reached prior to the murder-suicide. In those cases, the two couples, with all individuals over 80 years old, were married and had talked about their suicide plans. In one case, both 80-year-old participants signed the suicide note. In the other case, the offender stated in the suicide note that a mutual decision had been reached prior to the murder-suicide.

## Discussion

The most interesting finding of our study is the high mean age of the offenders (63 years) and victims (47 years). Other studies found an age range from 40 to 50 years among offenders [3]. The victims in this study were approximately 5.6 years younger than the offenders, which is in line with the literature. Most studies have shown that offenders are male in over 90% of the cases, and victims are usually female [1]. This is also in line with our study, which showed that all offenders were male and all victims were female except two male children.

Twelve out of 17 couples were married, and in 7 of the married couples, both individuals were over 80 years old. The most important motives for murder-suicides were disease and the loss of autonomy. This motive was identified in 6 cases, all involving individuals who were more than 80 years old. This might point towards deficiencies in the health care resources available for older patients or might also be a reflection of a change in demographics. Remarkably, “mercy killings” have seldom been reported in the literature [4, 6]. However, some authors have recently noted an increase in the age of the offender [10–12]. In at least 4 cases of murder-suicides involving older participants, there was evidence of a prior mutual agreement. Cases involving brutal causes of death, such as suffocation with a plastic bag or a cotton diaper or gunshot wounds, make a consensual murder-suicide implausible. One also has to consider the highly developed dependencies in long-term partnerships, which might make consensual murder-suicides very unlikely.

In four cases, separation from a partner was found to be the motive, and all the offenders were younger than 80 years old. This is the most commonly reported motive in the literature [1]. In one case that involved a 69-year-old father killing his wife and his two sons by carbon dioxide poisoning, possible financial motives were mentioned in his suicide note [13].

One offender killed his daughter. In the literature, this typology is mostly observed in “pseudoaltruistic” murder-suicides, and the offenders are generally female [6]. To our knowledge, the case described in this report is the only case of a murder-suicide in which a father killed his daughter and himself at the same time with one bullet.

In 8 out of 17 cases, gunshot wounds caused the deaths of the victims and the offenders. This was the most common cause of death, which is also in line with previous reports.

Since most persons involved in our study were over 80 years old, we suggest increasing efforts to prevent social isolation in older age groups. This is especially important during the current COVID-19 pandemic. Psychiatric diagnoses such as depression are possible risk factors for murder-suicides. Public awareness campaigns might help destigmatize psychiatric disorders [14]. Additionally, health care resources, especially for older persons, should be optimized. Since intoxication with various substances can also play an important role, healthcare professionals should be sensitized to the possible lethal consequences of their prescription practices, especially with regard to tranquilizers. A central issue should also be the prevention of domestic violence, which is an important risk factor for murder-suicides, especially those involving children [17].

Some limitations of this study include problems determining the motive for the murder-suicide due to a lack of, for example, further information such as newspaper articles or suicide notes. Since the motive is essential for the identification of a murder-suicide because of the primacy of the suicidal intention, the real number of murder-suicides was most likely higher than the 17 cases we identified. Some supposed double suicides in which both participants were under the influence of a drug might also be considered as murder-suicides because one participant might have actively administered the sedative to the other participant. Rosenbaum et al. [18] also suggested that there is often pressure applied by one participant in supposed double suicides. The differentiation between a double suicide and a murder-suicide was also described as problematic by Jensen et al. [19]. They found that double suicides often involved the use of less violent methods than in murder-suicides, and chronic disease was often identified as the motive [19]. Byard et al. [20, 21] also discussed the problems involved in retrospectively determining the motive behind a vehicle crash involving the deaths of all the family members. This type of incident might be a subcategory labeled “familial vehicular murder-suicide” [20, 21]. Another possible category is “murder-accidents”, which might be implicated in, for example, deaths related to burnt out houses [22]. Further possible categories are “aircraft-assisted pilot suicides” [23–28] and “suicide bombings” [17, 29], although whether these can be considered murder-suicides is controversial. We propose establishing the category of “consensual murder-suicides” [30] as a subcategory of murder-suicides to facilitate the analyses of those cases involving possible mutual agreement between the participants.

It is important to perform a psychopathological analysis in all potential cases of murder-suicide, even though no legal proceeding is instigated due to deaths of both the victim and the offender [1]. Such an analysis facilitates the further investigation of the motives and typology of murder-suicides. This is essential for developing prevention strategies. It is also important to establish a central register for murder-suicides, as there are for homicides and suicides, and historical archives to document previous murder-suicides [31].

Overall, our findings provide further information about the typology of murder-suicides, which might support the establishment of prevention strategies in the future.

## Key points

- 1. We identified 17 murder-suicides in Berlin in a time-period of 8 years between 2005 and 2013.
- 2. In seven cases, the victims and offenders were at least 80 years old.
- 3. The average age of the offenders was 63 years.
- 4. All of the 17 offenders were male and 90% of the victims were female.
- 5. Our study implicates the need for improving the existing health care structures, especially for older adults and people with depression.

## Data Availability

The datasets are available upon reasonable request.
